# Healthcare can learn from space exploration to champion disability inclusion

**DOI:** 10.1038/s43856-024-00577-w

**Published:** 2024-07-25

**Authors:** Farhan M. Asrar, Dana Bolles, Thu Jennifer Ngo-Anh

**Affiliations:** 1 University of Toronto, Toronto, Ontario Canada; 2 International Space University, Strasbourg, France; 3 National Aeronautics and Space Administration (NASA), Washington, DC USA; 4 European Space Agency (ESA), Noordwijk, The Netherlands

**Keywords:** Health services, Public health

## Abstract

Asrar et al. discuss the steps that the space sector is taking towards promoting equity, diversity, inclusion and accessibility, such as the world’s first parastronaut program. They propose that healthcare can learn from the space sector in enhancing disability inclusion and support for people, including healthcare workers, with disabilities.

## Teaser

People with disabilities, including healthcare professionals, encounter many obstacles. The space sector is taking steps towards promoting equity, diversity, inclusion and accessibility, including developing the world’s first parastronaut program. Here, we propose that healthcare can learn from space in enhancing disability inclusion.

Despite the growing emphasis on equity, diversity, inclusion, and accessibility (EDIA) in the workplace, significant progress remains to be achieved. Numerous obstacles and difficulties persist for individuals with disabilities while pursuing employment opportunities and accessing healthcare services^1,2^. The International Labour Organization (ILO) indicates much higher rates of unemployment in people with disabilities compared to those without disabilities, and that employees often assume those with disabilities are unable to work^1^.

Many challenges for healthcare professionals and medical students with disabilities also exist^1,3^. Studies have shown learners with disabilities applying for medical education are often excluded, devalued, and stereotyped of not being able to handle medical training^3^. A recent study concluded that medical schools systematically exclude people with disabilities from the medical field, and noted that applicants were even advised to hide their disability in order to reduce any bias^3^. A deaf medical resident was dismissed from her residency after being denied her request for accommodations for sign language interpreters^4^. Physicians and medical students with disabilities have also felt the lack of support from the medical system itself, with medical administrators hesitant to work with people with disabilities, and standards and criteria set in place that exclude those with disabilities^3,4^. Another publication on disability inclusion in academia alluded to ableism (prejudice and discrimination toward those regarded as disabled) being a major part of disability discrimination^5^. Additionally, there are often environmental barriers for both patients and physicians in wheelchairs, when clinics and buildings lack accessibility, or exam tables are non-adjustable or even the lack of exam room space limits a wheelchair from entering.

Such discrimination not only affects advancing careers but also limits diversity and inclusivity of healthcare teams. Consequently, this impedes the delivery of comprehensive and empathetic patient care, as well as having a diverse and representative workforce which is better informed to adequately address the needs of a varied patient population. Tackling this issue requires a cultural shift towards greater acceptance and support for healthcare workers with disabilities.

Developments in the space sector towards disability inclusion can serve as a model for healthcare and other disciplines globally. Historically, space exploration involved a narrow demographic, with limited inclusion of women, individuals of diverse racial and ethnic backgrounds, and those with disabilities. The astronaut selection process, space hardware, and spacesuits were initially developed for men without disabilities. However, in recent years, the space sector has been undergoing a transformative shift, increasingly evolving into a platform that champions diversity, inclusion, and breaking down barriers^6^. NASA’s Artemis missions will help further encompass equity, diversity, and inclusion with the first woman, first person of colour, and first non-American heading to the Moon’s orbit and, eventually, stepping on the Moon.

**Fig. 1:**
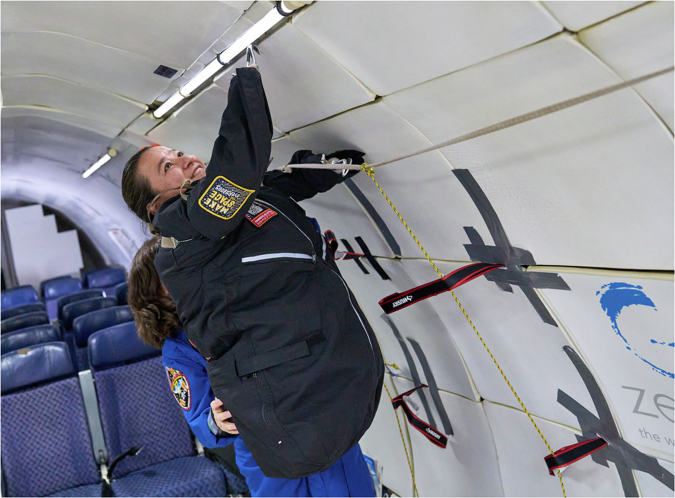
Dana Bolles (DB) from NASA and a mobility ambassador, onboard Mission: AstroAccess Flight One inaugural Zero-G flight. Seen with astronaut Dr. Cady Coleman. Credit: Al Powers from Zero Gravity Corporation. The authors affirm that informed consent for publication of this image was received from DB and Dr. Cady Coleman.

While the formal NASA astronaut selection process began to select more diverse crews as early as 1978, it wasn’t until very recently that we’ve seen focus on exposing the disability community to the possibilities of space exploration, with a number of recent initiatives promoting greater inclusion of people with disabilities. In 2017, the Kid’s Weightless Dreams campaign organized by Novespace and Reves de Gosse enabled disabled children to experience weightlessness and conduct science demonstrations^7^. In 2021, Mission AstroAccess (MAA), an organization dedicated to promoting disability inclusion in space exploration and in science, technology, engineering, and maths (STEM), flew its first crew of 12 disability ambassadors (including this publication’s co-author DB) on a zero gravity flight^8^ (Fig. 1).

In 2021, as a world first, the European Space Agency (ESA) introduced the Parastronaut Feasibility Project, that opened doors to selecting astronauts with disabilities^9^. Parastronaut is a term used by the European Space Agency to describe an astronaut with a physical disability. The selection process began in 2022 and Dr. John McFall, an accomplished physician and paralympic athlete, was selected as the world’s first parastronaut. The study is currently assessing the conditions for parastronauts to live and work in space. This project also challenges the norms by aiming to adapt technology, training, and spacecraft design to accommodate a broader range of physical abilities^9^. The findings of this study could also improve healthcare support and delivery for individuals with disabilities on Earth. That is, various technological and innovative solutions being developed for parastronauts would be applicable to assistive devices, and other adaptive equipment that can improve quality of life of those with disabilities on Earth. Research into the health and physical wellbeing of parastronauts can also lead to medical advances to assist people with disabilities. By studying common health concerns in space such as radiation exposure, bone loss, and muscle atrophy, amongst others, we gain further insight into how such conditions can impact those with disabilities. Additionally, the increased awareness of the parastronaut program could lead to societal and systemic changes towards increased diversity and inclusion of those with disabilities which, in turn, can reduce stigma, increase opportunities, and inspire policy changes and advocacy to promote greater collaboration between health professionals, STEM leaders and disability advocates.

Another interesting concept that the experience of parastronauts will help us better understand is whether a physical disability would be a barrier or perhaps even a benefit in space and other environments. Generally, space is a disabling environment: over time, any person outside of the confines of Earth will experience impairment through exposure to radiation, microgravity, and other features of the environment that result in health issues including bone loss, muscle deterioration, changes in the immune system, and other long-term effects we are only beginning to understand. Thus, would someone with a physical disability such as missing lower limb(s) have limitations over astronauts without a disability in space, where all float to move rather than using their legs? NASA conducted experiments in the 1950s (known as Gallaudet 11) that involved 11 deaf participants whose vestibular systems of their inner ears were damaged, making them immune to motion sickness. These findings alluded to deaf participants being more adaptable to foreign gravitational environments^10^. Such an analogy would help to underscore the potential benefits that health professionals with disabilities bring to the healthcare system, particularly in supporting and treating patients with disabilities. Having lived an experience of being disabled, healthcare professionals with disabilities bring a unique perspective, empathy and better understanding of the challenges that a patient with disabilities is going through. Furthermore, living with a disability often results in that person being a more creative problem solver. Since the person cannot typically do activities in the same way as a non-disabled person, they must think outside the box and figure out how to address it within their abilities.

By drawing parallels between the successes and challenges faced by parastronauts and physicians with disabilities, we can better understand the value and contributions healthcare professionals with disabilities bring to the health system. The achievements of parastronauts can also serve as an inspiration for healthcare professionals with disabilities, in addition to all people with disabilities interested in careers in STEM.

Ultimately, space exploration is transcending historical challenges, emerging as a catalyst for EDIA and serving as an inspiration to encourage greater inclusivity in STEM. Initiatives like the parastronaut program not only break barriers, but also redefine the possibilities of inclusivity, shaping a future where careers in space and STEM are open to all. Healthcare leaders and decision-makers can draw inspiration from the advancements made in the space sector to overcome the norms. From a time when individuals with disabilities might have been excluded, the space sector now spearheads initiatives actively inviting them to participate as astronauts/parastronauts. This serves as a compelling example for healthcare to similarly adapt, by integrating technology, upgrading equipment; refining training methods, standards and selection criteria; and creating inclusive environments to better accommodate people with disabilities.

## Reporting summary

Further information on research design is available in the Nature Portfolio Reporting Summary linked to this article.

### Supplementary information

Peer Review File

Reporting Summary
